# Sutures after Abdominal Incisions

**Published:** 1900-06-09

**Authors:** 


### SUTURES AFTER ABDOMINAL INCISIONS.

At the recent meeting of the American Surgical Association[1] a discussion arose as to the method to be employed in closing the abdominal wound after cœliotomy. Dr. M. H. Richardson placed great stress on the advantages of the through and through suture over suture *en etage*, claiming, first, that it left no blind spaces; and, second, that it was very quickly done. The materials which he employed were silk, silver wire, and silkworm gut. Of 2,000 cœliotomies at the Massachusetts General Hospital which were sutured through and through, and which healed by primary union, but 12 returned with recurrence. Buried sutures should be non-absorbable, for they should add to the strength of the tissues in which they lie, and silk was to be preferred. He emphasised the point that accurate approximation layer by layer was not necessary for strong union. Dr. Weir pointed out the necessity of leaving sutures somewhat loose, urging that constriction produced a slough, which gave a nidus for the skin germs which might be found in every wound. Dr. Coley endorsed this, and laid emphasis upon the necessity of reducing the chances of infection to a minimum. He claimed that catgut could be absolutely sterilised; and that, while prior to the use of rubber gloves at operations there had been 6 per cent. of infection in hernia cases at the Hospital for the Relief of the Ruptured and the Crippled, there had been but one infection in the last 150 cases since gloves had been used. Other speakers agreed as to the necessity for not tying sutures too tightly, but doubt was expressed as to buried non-absorbable sutures continuing for any length of time to add strength, it being held by some of the speakers that after a few days they could do no good.

[1] New York Med. Rec., May 19.

